# Estrogen receptor α-coupled Bmi1 regulation pathway in breast cancer and its clinical implications

**DOI:** 10.1186/1471-2407-14-122

**Published:** 2014-02-24

**Authors:** Huali Wang, Haijing Liu, Xin Li, Jing Zhao, Hong Zhang, Jingzhuo Mao, Yongxin Zou, Hong Zhang, Shuang Zhang, Wei Hou, Lin Hou, Michael A McNutt, Bo Zhang

**Affiliations:** 1 Department of Pathology, Health Science Center of Peking University, 38 Xueyuan Road, Haidian District, Beijing 100191, China; 2 Department of Pathology, Peking University First Hospital, Beijing 100034, China

**Keywords:** Bmi1, Estrogen receptor α, p16^INK4a^, Cyclin D1, Breast cancer

## Abstract

**Background:**

Bmi1 has been identified as an important regulator in breast cancer, but its relationship with other signaling molecules such as ERα and HER2 is undetermined.

**Methods:**

The expression of Bmi1 and its correlation with ERα, PR, Ki-67, HER2, p16^INK4a^, cyclin D1 and pRB was evaluated by immunohistochemistry in a collection of 92 cases of breast cancer and statistically analyzed. Stimulation of Bmi1 expression by ERα or 17β-estradiol (E2) was analyzed in cell lines including MCF-7, MDA-MB-231, ERα-restored MDA-MB-231 and ERα-knockdown MCF-7 cells. Luciferase reporter and chromatin immunoprecipitation assays were also performed.

**Results:**

Immunostaining revealed strong correlation of Bmi1 and ERα expression status in breast cancer. Expression of Bmi1 was stimulated by 17β-estradiol in ERα-positive MCF-7 cells but not in ERα-negative MDA-MB-231 cells, while the expression of Bmi1 did not alter expression of ERα. As expected, stimulation of Bmi1 expression could also be achieved in ERα-restored MDA-MB-231 cells, and at the same time depletion of ERα decreased expression of Bmi1. The proximal promoter region of *Bmi1* was transcriptionally activated with co-transfection of *ERα* in luciferase assays, and the interaction of the *Bmi1* promoter with ERα was confirmed by chromatin immunoprecipitation. Moreover, in breast cancer tissues activation of the ERα-coupled Bmi1 pathway generally correlated with high levels of cyclin D1, while loss of its activity resulted in aberrant expression of p16^INK4a^ and a high Ki-67 index, which implied a more aggressive phenotype of breast cancer.

**Conclusions:**

Expression of Bmi1 is influenced by ERα, and the activity of the ERα-coupled Bmi1 signature impacts p16^INK4a^ and cyclin D1 status and thus correlates with the tumor molecular subtype and biologic behavior. This demonstrates the important role which is played by ERα-coupled Bmi1 in human breast cancer.

## Background

Breast cancer which is currently the most common malignant tumor in females worldwide, shows characteristic heterogeneity that has a genetic or molecular basis. Thus far at least five molecular subtypes of breast cancer have been defined that include Luminal-A, Luminal-B, Luminal-B-HER2, HER2-enriched and basal like. Definition of these subtypes has allowed treatment to be tailored directly for each type in breast cancer, and marked progress has been made in improving patient survival rate [1]. However, varying sensitivity to treatment and resistance to endocrine or targeted therapy which may be found de novo or may be acquired still presents a therapeutic challenge. Much effort is still needed to completely characterize all the molecular details which may be related to therapeutic targets in breast cancer.

As a hormonally driven tumor, breast cancer is closely associated with estrogen and its α receptor (ERα), in either the process of carcinogenesis or in tumor biology. Up to 70% of breast cancers show ERα expression, and two-thirds of ERα-positive breast carcinoma patients respond to treatment with anti-estrogen therapy [2-4], while breast cancer lacking ERα expression does not benefit from endocrine treatment. Nevertheless, many patients with ERα positive cancer are unresponsive to endocrine therapy, and all patients with advanced disease eventually develop resistance to the therapy [2,5]. ERα-associated signaling has therefore become a topic of significant interest in the battle against breast cancer. Like other steroid receptors, ERα can directly activate its target genes such as PR and cyclin D1 through an interactive element (ERE, estrogen responsive element) [6]. In a recent study, ERα has been shown to cross talk with other growth factor pathways (non-genomic activity) [6]. In addition to genetic and protein interaction, epigenetic mechanisms of ERα regulation have also received attention in recent years. Silencing or reactivation of ERα by epigenetic regulation has been demonstrated in cultured breast cancer cells [7]. At the same time, the expression of HOXB13 or CDK10 regulated by promoter methylation affects ERα status [8,9]. Moreover, epigenetic modification has been documented in breast cancer.

*Bmi1* (Bmi1 polycomb ring finger oncogene) which encodes a polycomb ring finger protein, was originally cloned as a *c-myc* cooperating oncogene in murine lymphoma [10]. It has subsequently been identified as a transcriptional repressor belonging to the polycomb group (PcG) proteins, and is also a key factor in the polycomb repressor complex 1 (PRC1), which serves as an important epigenetic regulatory complex for modulation of chromatin remodeling [11]. To date, many PRC1 target genes have been identified including homeobox (*HOX*) genes and *p16*^*INK4a*^, whose promoters contain interactive elements which bind directly to *Bmi1*[12]. A striking finding in recent studies is that the activity of Bmi1 is indispensable for cell survival and self-renewal of stem cells or cancer stem cells [13-15]. Over-expression of Bmi1 has been found in a large number of human cancers, and a set of 11 genes which make up the Bmi1 signature has been defined in colorectal, breast, lung and prostate cancers [16-18]. Bmi1 expression in breast cancer has also been found to be associated with other tumor genes [19-21] and *in vitro* models have demonstrated Bmi1 is required for metastasis of breast cancer [22]. However, there has been no demonstration of any relationship of Bmi1 with other significant factors in breast cancer such as ERα, PR, HER2 and Ki-67.

In this study, we at first identified a strong correlation of ERα status with Bmil expression in a collection of breast cancer tissues, and we then demonstrated the positive regulatory role ERα may play in transcriptional expression of the Bmi1 gene. The ERα-coupled Bmi1 regulatory pathway was subsequently evaluated with regard to its down-stream genes such as *p16*^*INK4a*^ and *cyclin D1* and clinic-pathological features in breast cancer. Results strongly suggest the ERα-coupled Bmi1 regulatory pathway may be one of the main regulatory mechanisms in breast cancer, whose activity determines the down-stream gene status of *p16*^*INK4a*^ and *cyclin D1*, and consequently impacts the biologic behavior of breast cancer.

## Methods

### Ethics statement

Paraffin-embedded archival breast cancer tissues were obtained from the Pathology Department of Peking University Third Hospital. This study was conducted after receiving approval from the Peking University Health Science Center Institutional Review Board (IRB). Primary tumor samples were all collected from archival tissues with deletion of all patient identifiers from the retrospective clinical data used in our study. Sample and data collection were approved for informed consent waiver by the IRB.

### Tissue specimens

Tumor samples were obtained from radical mastectomies in 92 cases of invasive breast carcinoma confirmed by histopathology in the Pathology Department of Peking University Third Hospital. All cases were scored histologically as grade I, II and III, according to the Nottingham grading criteria which includes extent of formation of glandular lumina, nuclear atypia and the mitotic index. The TNM classification classes T1 to T4 were used to evaluate the tumor size (T1: ≤ 2 cm,T2: >2 cm but ≤ 5 cm, T3: > 5 cm and T4: tumor of any size, with direct extension to chest wall or skin). The clinical characteristics of the patients are summarized in Additional file 1: Table S1.Tumor tissues were fixed in 4% neutral–buffered formaldehyde solution (pH 7.0) and were routinely processed for paraffin embedding. Sections of 4 μm were used for immunohistochemistry staining.

### Immunohistochemistry (IHC)

Paraffin-embedded sections were hydrated with serial treatment with xylene and graded alcohols. Endogenous peroxidase activity was blocked with 0.3% hydrogen peroxide for 60 min. Antigen retrieval was carried out by heating at 95°C in 2 × 10^−2^ M citrate buffer (pH 6.0) or 10^−3^ M EDTA buffer (PH 8.0) for 20 min. After blocking with horse serum (1:100), sections were incubated with primary antibody (Additional file 1: Table S2) diluted with PBS to various concentrations at 4°C overnight, followed by washing in PBS. Antibody reactions were colorized with the Dako REAL™ EnVision™ Detection System (Dako, Glostrup, Denmark). Sections were counterstained with Mayer’s hematoxylin. Positive and negative (primary antibody replaced by PBS) controls were included for all staining procedures.

### Staining evaluation

Immunohistochemistry (IHC) staining was evaluated independently by two pathologists blinded from the clinical data. Bmi1, cyclin D1 and pRB generally showed nuclear staining in a diffuse pattern, and a negative reaction was defined as absence of staining or occasional positive cells which were less than 5% of the total tumor cells. ERα and PR were scored as positive if at least 1% of tumor cell nuclei were positive [23], but in our collection of specimens, a positive reaction typically had more than 20% positive cells. HER2 was scored by accepted criteria where intensity and completeness of membrane staining were evaluated as previously described [24]. Ki-67 values were calculated as the percent of positively stained cells in at least three randomly selected high power fields (× 40 objective) [25]. The aberrant expression of p16^INK4a^ (+) in cancer cells was defined by cytoplasmic staining with or without nuclear staining, distributed either multifocally (10%-49% of cancer cells) or diffusely (≥ 50% cells). Negative staining (―) was defined as no staining in any cells, or no more than only occasional positive cells (less than 5%). The subtypes in immunohistochemistry were classified according to the reference and the cutoff of Ki-67 for determination of Luminal-A or -B is 14% [1].

### Statistical analysis

All data were analyzed with SPSS statistical software (Version 13.0, Chicago, IL, USA). Relationships between tumor markers and other parameters were analyzed using the χ^2^-test, Pearson Chi-square test, Fisher’s exact test or Student’s t test. *P*-values of less than 0.05 were considered to be statistically significant and tests were two tailed.

### Cell culture and treatment

Human breast carcinoma MCF-7 and MDA-MB-231 cell lines were maintained in DMEM (GIBCO, Carlsbad, CA, USA) supplemented with 10% FBS (HyClone, Logan, UT) at 37°C. For steroid treatment, cells were first cultured in phenol-free DMEM (GIBCO) containing 10% double charcoal-stripped FBS (Bioind, Kibbutz Beit Haemek, Israel) for 72 h and then incubated with 10^−8^ M 17β-estradiol (E2) (Sigma, St Louis, MO, USA) or 10^−6^ M 4-Hydroxytamoxifen (4-OHT) (Sigma) dissolved in ethanol, or with ethanol only (as a vehicle control) for indicated lengths of time.

### Western blot

Total protein samples from cell lysates were resolved on SDS-polyacrylamide gels of different concentrations and transferred onto nitrocellulose membranes (Amersham Pharmacia Biotech, Uppsala, Sweden). After blocking with 5% nonfat milk for 60 min, membranes were incubated with appropriate primary antibodies (Additional file 1: Table S2) at 4°C overnight, followed by incubation with alkaline phosphatase-conjugated secondary antibody, and were visualized using NBT/BCIP (Promega, Madison, WI, USA). Densitometry was performed with Image J (1.42q Software, NIH Public Domain).

### Plasmids and transfection

Human *Bmi1* [GenBank: NM_005180] was amplified with primers 5′-GCAGATCTATGCATCGAACAACGAG-3′ (forward) and 5′-GCGTCGACTCAACCAGAAGAAGTTG-3′ (reverse). Total RNA was isolated from cells with Trizol reagent according to the manufacture’s protocol (Invitrogen, Carlsbad, CA, USA) and was reversely transcribed into cDNA with AMV reverse transcriptase (Promega). The PCR product was digested with appropriate restriction enzymes and subcloned into multiple cloning sites of the pcDNA3.1/HisC vector (Invitrogen) and sequenced, generating pcD-Bmi1. The pcDNA3.1-ERα expression plasmid was a gift from Dr. Yongfeng Shang.

By using Lipofectamine 2000 reagent (Invitrogen), MCF-7 cells were transiently transfected with pcD-Bmi1. MDA-MB-231 cells were transfected with pcDNA3.1-ERα (or empty vector) following the manufacture’s instruction and selected in G418 (0.6 mg/ml). The stable clones which were generated were designated as 231/ERα and 231/vec, respectively.

### Gene silencing with small interfering RNAs (siRNAs)

Three pairs of double-stranded siRNAs were synthesized (GenePharma, Shanghai, China) based on the *ERα* mRNA sequence [GenBank: NM_000125.3], including siRNA1 sense-5′-CAGGCCAAAUUCAGAUAAUTT-3′, and antisense-5′-AUUAUCUGAAUUUGGCCUGTT-3′; siRNA2: sense-5′-GAGGGAGAAUGUUGAAACATT-3′, and antisense-5′-UGUUUCAACAUUCUCCCUCTT-3′; and siRNA3 sense -5′-GGUCCACCUUCUAGAAUGUTT-3′, and antisense-5′-ACAUUCUAGAAGGUGGACCTT-3′. 4 × 10^5^ cells in 6-well plates were transiently transfected with 100 pmol *ERα* siRNA using Lipofectamine 2000 reagent following the manufacture’s instruction. These experiments were carried out independently three times.

### Real time RT-PCR

Total RNA was isolated from cells with Trizol reagent according to the manufacture’s protocol (Invitrogen, Carlsbad, CA, USA) and was reversely transcribed into cDNA with AMV reverse transcriptase (Promega). Real-time PCR was set up with the Stratagene Mx3000p (Agilent Technologies, Santa Clara, CA, USA) by using Brilliant® II SYBR Green QPCR Master Mix (Agilent Technologies). PCR was performed at 95°C for 15 s and 60°C for 60 s for 40 cycles. Primer sequences were as follows: ERα, 5′-TGCCCACTACTCTGGAGAAC-3′(forward) and 5′-CCATAGCCATACTTCCCTTGTC-3′(reverse); Bmi1, 5′-AATTAGTTCCAGGGCTTTTCAA-3′(forward) and 5′-CTTCATCTGCAACCTCTCCTCTAT- 3′(reverse); p16^INK4a^, 5′-GCTGCCCAACGCACCGAATA-3′(forward) and 5′-ACCACCAGCGTGTCCAGGAA-3′(reverse); β-actin, 5′-ATCATGTTTGAGACCTTCAACA-3′(forward) and 5′-CATCTCTTGCTCGAAGTC-3′(reverse). The β-actin from the same extracts was used as an internal control. The amount of ERα, Bmi1 and p16^INK4a^ were normalized to the β-actin value. Data were calculated from the mean of three experiments.

### Reporter construction and luciferase assay

**Figure 1:**
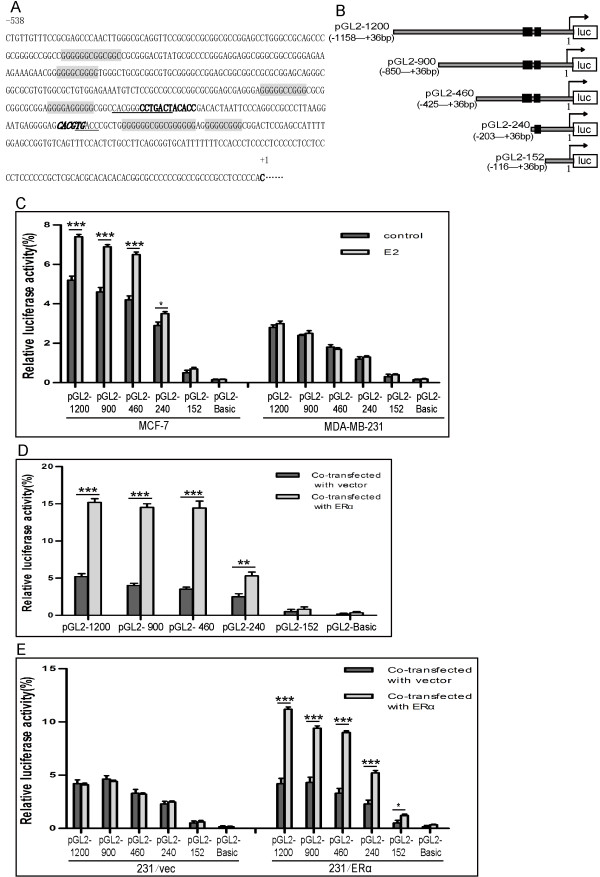
**Effects of ERα on the transcriptional activity of Bmi1 promoter. (A)** The composition of the Bmi1 core promoter. The transcription element E-box is in italics, AP-1 is in boldface, several Sp-1 s are in the shadow box, and the putative ERα response elements (ERE) are underlined. +1 indicates the transcription start. **(B)** Luciferase reporter construction. A series of reporters including pGL2-1200, pGL2-900, pGL2-460, pGL2-240 and pGL2-152 were constructed spanning the sequence +36 nt to −1158 nt of the Bmi1 promoter, and the two putative EREs were in black box. **(C)** The transcriptional activity of the Bmi1 gene promoter in ERα-positive or –negative breast cancer cells. MCF-7 and MDA-MB-231 cells were cultured in phenol red free medium containing 10% charcoal stripped FBS and transiently transfected with 200 ng each of empty pGL2-basic, pGL2-1200, pGL2-900, pGL2-460. pGL2-240 or pGL2-152 in the absence or presence of 10^−8^ M E2, respectively. Cells were harvested 48 h after transfection and assayed for luciferase activity. **(D)** The transfection of ERα enhanced transcriptional activity of the Bmi1 promoter. MCF-7 cells were co-transfected with 200 ng each of reporter plasmids and 200 ng of ERα expression plasmid (pcDNA3.1-ERα) or pcDNA3.1 empty vector. Cells were harvested 48 h after transfection and assayed for luciferase activity. **(E)** The reactivation of Bmi1 promoter in ERα-restored ERα-negative cells. ERα-restored MDA-MB-231 cells (231/ERα) or their control 231/vec cells were transfected with 200 ng of each of the reporter plasmids. The relative luciferase activity values are corrected for co-transfected Renilla activity. And the experiments were repeated at least three times independently and all data are shown by bars as means ± SD (^*^P < 0.05, ^**^P < 0.01, ^***^P < 0.001 when compared with the control groups, respectively).

Genomic DNA was prepared using standard molecular techniques and was used as a template for amplification of the *Bmi1* promoter [GenBank: NC_000010.10.3] with three different pairs of primers as follows: region 1 (−1158 ~ +36) sense sequence 5′-CTTCAGCTGAACCACCGTTTGTG-3′ and antisense sequence 5′-GCCAAGCTTCTGCCTCTCATACTACG-3′; region 2 (−850 ~ +36) sense sequence 5′-GTTCAGCTGCTAGATAGGAGTAGTGTG-3′ and antisense sequence 5′-GCCAAGCTTCTGCCTCTCATACTACG-3′; region 3 (−203 ~ +36) sense sequence 5′-GTTCAGCTGCCCTTAAGGAATGAGG-3′ and antisense sequence 5′-GCCAAGCTTCTGCCTCTCATACTACG-3′; and region 4 (−116 ~ +36) sense sequence 5′-GTTCAGCTGTCAGTTTCCACTCTG-3′ and antisense sequence 5′-GCCAAGCTTCTGCCTCTCATACTACG-3′. PCR products were digested with appropriate restriction enzymes and subcloned into multiple SmaI-Hind III cloning sites on the pGL2-Basic plasmid (Promega) and sequenced, generating pGL2-1200, pGL2-900, pGL2-460, pGL2-240 and pGL2-152 (Figure 1B).

Transfection was performed in 24-well plates (1 × 10^5^ cells/per well) using Lipofectamine 2000 reagent with 200 ng of reporter (or pGL2-basic) and 2 ng of pRL-SV-Renilla reference vector (Promega). Alternatively, in some experiments 200 ng pcDNA3.1-ERα with 200 ng of reporter (or pGL2-basic) and 2 ng of pRL-SV-Renilla reference vector were co-transfected. Protein lysates were prepared from post-transfected cells, and luciferase activities were measured with the Dual-Luciferase Reporter Assay System (Promega) using a MicroBeta TriLux Liquid Scintillation and Luminescence Counter (Perkin-Elmer, Waltham, MA, USA). Firefly luciferase activity was normalized to Renilla luciferase activity and presented as a ratio (relative luciferase activity). All experiments were performed independently at least three times.

### Chromatin immunoprecipitation (ChIP)

MCF-7, MDA-MB-231 and 231/ERα cells were held in steroid starvation for 3 days and then treated with 10^−8^ M E2 or vehicle (12 h) at 80% confluence. ChIP was performed as previously described [26]. Briefly, 5 × 10^6^ cells per ChIP assay were cross-linked with 1% formaldehyde for 10 min at 37°C and then quenched with 125 mM glycine. Cells were washed with cold PBS and scraped into PBS with protease inhibitors (Roche, Indianapolis, IN, USA). Cell pellets were resuspended in ChIP lysis buffer (1% SDS, 10 mM EDTA, 50 mM Tris–HCl. pH 8.1) and sonicated with an Ultrasonic Homogenizer (Cole-Parmer, Chicago, IL, USA) to produce sheared chromatin with an average length of 500 bp. The sheared chromatin was subjected to a clarification spin and the supernatant was then used for ChIP or reserved for analysis of “Input”. Anti-ERα antibody (Epitomics) was used and normal rabbit IgG (Sigma) was used as negative control. Primers for the ChIP-PCR assay were as follows: ChIP primers (−327 ~ −172) for sense: 5′-CGTGTGGCGCTGTGGAGAAATGTCT-3′ and antisense: 5′-GGGTCACGTGCTCCCCTCATTCCTT-3′; ChIP negative control primers (−2647 ~ −2523) sense: 5′-GTGGAAAGTAGAGCCATTCT-3′ and antisense: 5′-AAACATCCGTTATATGAGGG-3′.

## Results

### The expression of Bmi1 strongly correlated with ERα status in breast cancer

**Figure 2:**
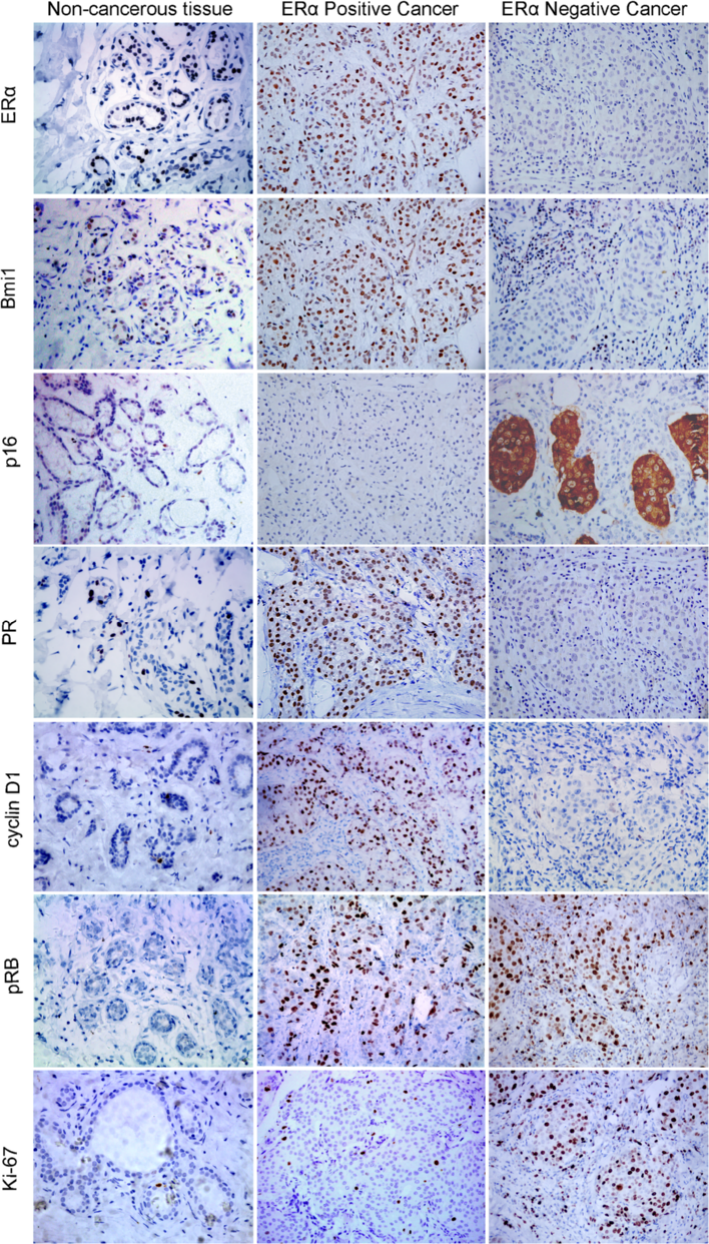
**Expression of ERα, Bmi1, p16**^**INK4a**^**, PR, cyclin D1, pRB and Ki-67 in breast carcinoma.** The left column: the representative images for staining of ERα, Bmi1, p16^INK4a^, PR, cyclin D1, pRB and Ki-67 in non-cancerous breast tissue. The middle column: the representative images for ERα positive breast cancer with Bmi1 positive and p16^INK4a^ negative. The various positive staining of PR, cyclin D1, pRB and low Ki-67 index are presented. The right column was representative images for ERα negative breast cancer with negative Bmi1 but diffuse staining of p16^INK4a^. The various staining of PR, cyclin D1, pRB and high Ki-67 index are presented, respectively. (Hematoxylin /DAB, × 400).

Expression of Bmi1 was found in most non-neoplastic tubular epithelial cells in breast tissue, and was also found in a large proportion of breast cancer (79.35%, 73/92) by immunohistochemistry (Figure 2). Positive staining for Bmi1 was analyzed for comparison with other routine markers of breast cancer including ERα, PR, HER2, and Ki-67. The extent of positive staining for Bmi1 overlapping ERα-positivity was striking (98.33%, 59/60), and this was much less extensive overlap in the ERα-negative group (43.75%, 14/32). Loss of Bmi1 expression was extraordinarily rare in the ERα-positive group (1.67%, 1/60) as compared to the ERα-negative group (56.25%, 18/32). Similarly, ERα positivity was found in 80.82% (59/73) of the Bmi1 positive group and in 5.26% (1/19) of the Bmi1 negative group. These data indicate that the expression of Bmi1 is positively correlated with estrogen receptor α status (*P <* 0.0001) (Table 1). And expectedly, Bmi1 showed similar rates of positivity in both Luminal-A (100.00%, 28/28) and Luminal-B (96.15%, 25/26) (*P =* 0.481) (Table 2). To further evaluate expression of Bmi1, its target gene p16^INK4a^ was analyzed in both Bmi1-positive and negative groups with immunohistochemistry, and staining results confirmed Bmi1 status (see ERα-coupled Bmi1 regulatory signature in breast cancer in Results).

**Table 1:** **The correlation of Bmi1 or p16**^**INK4a **^**expression with other commonly used markers of breast cancer**

	**Bmi1**		***P *** **value**	**p16**^**INK4a**^		***P *** **value**
	**–**	**+**		**–**	**+**	
**ERα**			**<0.0001**			**<0.0001**
–	18	14		9	23	
+	1	59		56	4	
**PR**			**<0.0001**			**<0.0001**
–	17	16		11	22	
+	2	57		54	5	
**Ki-67**			**<0.0001**			**<0.0001**
0–13%	3	35		33	5	
14–29%	1	15		15	1	
30–49%	4	14		11	7	
50–100%	11	9		6	14	
**Bmi1**			**--**			**<0.0001**
–	–	–		1	18	
+	–	–		64	9	

Bold face representing significant data (P<0.05).

**Table 2:** **The aberrant expression of p16**^**INK4a **^**or Bmi1 in molecular subtypes of breast cancer**

**Subtypes**	**p16**^**INK4a**^		***P *** **value**	**Bmi1**		***P *** **value**
	**-**	**+**		**-**	**+**	
LA	28	0	**<0.0001**	0	28	**<0.0001**
LB	22	4		1	25	
LHP	8	0		0	8	
HP	5	11		7	9	
TNBC	2	12		11	3	
Total	65	27		19	73	

*Abbreviations*: *LA* Luminal-A, *LB* Luminal-B, *LHP* Luminal-HER2-Positive, *HP* HER2-Positive (HER2-enriched), *TNBC* Triple Negative Breast Cancer.

Bold face representing significant data (P<0.05).

Since Bmi1 and ERα are both transcription regulators, this marked overlap of expression suggested that Bmi1 and ERα could mutually regulate each other in a direct way. At the same time, detailed analysis showed that the rate of Bmi1 positivity in the ERα positive group was 98.33% (59/60), which was much higher than the positive rate of ERα in the Bmi1 positive group (80.82%, 59/73). In addition, in view of the fact that Bmi1 is a transcription repressor, it seemed likely that ERα positively regulates the expression of Bmi1.

Taken together, these data suggested there is a correlation between the expression of Bmi1 and ERα status and raised the possibility that ERα affects Bmi1 expression.

### ERα specifically regulates the expression of Bmi1 in breast cancer cells

**Figure 3:**
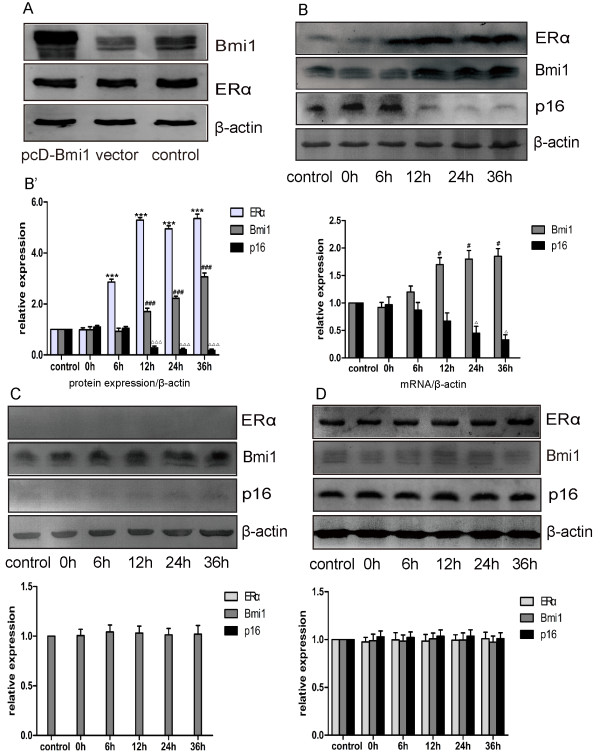
**Expression of Bmi1 was stimulated by E2 in ERα-positive breast cancer cells. (A)** Effect of Bmi1 ectopic expression on ERα protein in MCF-7 cells. MCF-7 cells were transiently transfected with Bmi1 (pcD-Bmi1), an empty vector, or transfection reagent (control). Cells were collected after 48 h of transfection and analyzed for Bmi1, ERα and β-actin expression with Western blot. This image represents one of three experiments. **(B-D)** Expression of Bmi1 was stimulated by E2 in ERα-positive breast cancer cells. ERα-positive MCF-7 **(B)** and ERα-negative MDA-MB-231 **(C)** cell lines were cultured in phenol red free medium containing 10% charcoal striped FBS for 72 h and 10^−8^ M E2 was added. At indicated time points, cells were collected and analyzed for Bmi1, ERα, p16^INK4a^ and β-actin expression by Western blot and real time RT-PCR (**B’**, right panel). **(D)** MCF-7 cells were treated with 10^−6^ M OHT in the presence of E2 and Western blot was performed. β-actin was used as loading control. Quantitative analyses of ERα, Bmi1 and p16^INK4a^ are presented. All data were obtained from three independent experiments and are shown by bars as means ± SD (^*,# or △^P < 0.05, ^**,## or △△^P < 0.01, ^***,### or △△△^P < 0.001 when ERα, Bmi1 and p16^INK4a^ were compared with the control group, respectively).

These data raised the possibility that ERα influences Bmi1 expression, however, to rule out the possibility that Bmi1 affects ERα expression, we repeatedly transiently transfected MCF-7 cells with ectopic Bmi1, and confirmed that introduction of Bmi1 has no effect on the expression of ERα (Figure 3A).

To determine whether Bmi1 is regulated by ERα, two breast cancer cell lines, ERα-positive MCF-7 and ERα-negative MDA-MB-231, were selected and treated with 10^−8^ M ERα ligand E2. In the presence of E2 (10^−8^ M), the expression of Bmi1 in MCF-7 cells was enhanced in a time-dependent manner, peaking at 12 h and persisting for at least 36 h. At the same time, the level of p16^INK4a^ declined over a time course similar to that of Bmi1 (Figure 3B). Conversely, the expression of Bmi1 in ERα negative MDA-MB-231 cells showed no significant response to the addition of 10^−8^ M E2 (Figure 3C). Moreover, the E2-stimulated expression of Bmi1 and consequent suppression of p16^INK4a^ in MCF-7 cells was antagonized by the antagonist OHT at 10^−6^ M (Figure 3D).

**Figure 4:**
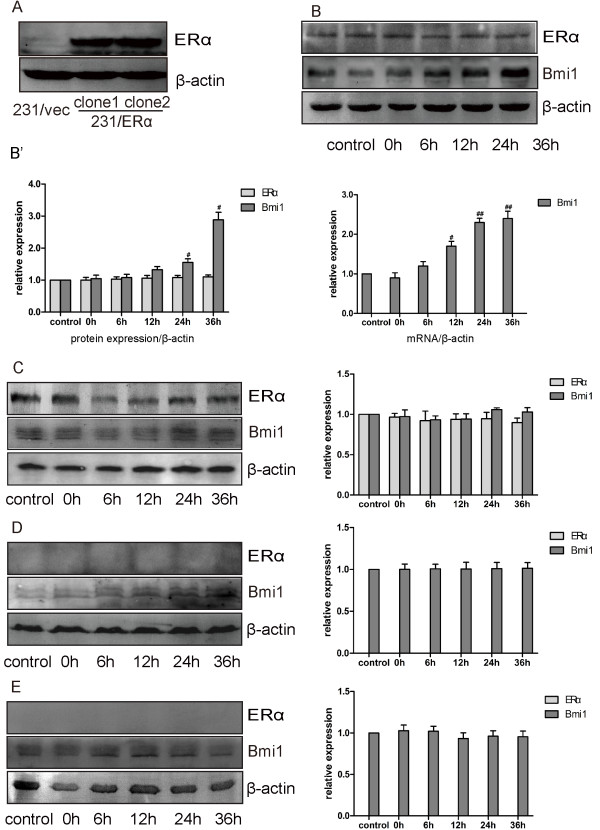
**Expression of Bmi1 was stimulated by E2 in ERα-restored breast cancer cells. (A)** 231/ERα and 231/vec were generated by stable transfection of MDA-MB-231 cells by ERα or empty vector, respectively. **(B)** 231/ERα and **(D)** 231/vec cells were stimulated with 10^−8^ M E2, and 10^−6^ M OHT was added at the same time, **(C and E)**. At indicated time, cells were collected and analyzed for Bmi1, ERα and β-actin expression by Western blot and real time RT-PCR (**B’**, right panel). β-actin was used as loading control. Quantitative analyses of ERα, Bmi1 and p16^INK4a^ are presented. All data were obtained from three independent experiments and are shown by bars as means ± SD (^#^P < 0.05 when Bmi1 was compared with the control group).

To further evaluate stimulation of Bmi1 expression by ERα, ectopic ERα (pcDNA3.1-ERα) was stably introduced into the ERα-negative MDA-MB-231 cells (Figure 4A). As a result, the ERα-restored MDA-MB-231 cells (231/ERα) displayed elevation of Bmi1 expression in a time dependent manner in the presence of 10^−8^ M E2 (Figure 4B), which was also inhibited by the addition of 10^−6^ M OHT (Figure 4C). Conversely, the expression of Bmi1 in ERα negative 231/vec cells showed no significant response to the addition of 10^−8^ M E2 (Figure 4D) and 10^−6^ M OHT (Figure 4E).

**Figure 5:**
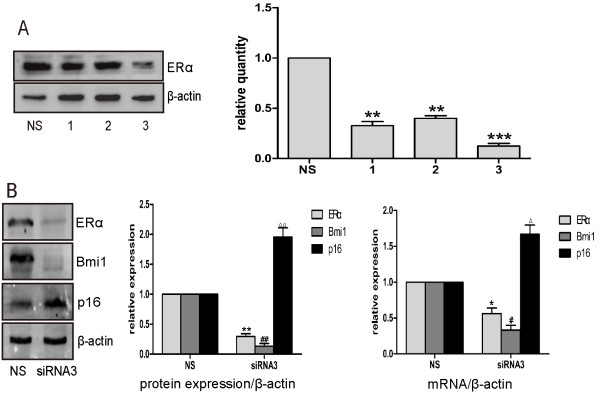
**Depletion of ERα decreased the level of Bmi1 and its E2 response. (A)** MCF-7 cells were transiently transfected with three sets of siRNAs specific for ERα (siRNA1, siRNA2, siRNA3) or with a control smart pool siRNA (NS), and 72 h after transfection cells were analyzed by Western blot (left panel). Quantitative analyses of ERα protein expression by the three siRNAs are presented (right panel). **(B)** MCF-7 cells were transiently transfected with siRNA3, and Bmi1, ERα and p16^INK4a^ were analyzed by Western blot (left panel) and real time RT-PCR (right panel). β-actin was used as loading control. Quantitative analyses of ERα, Bmi1 and p16^INK4a^ are presented (middle panel). Right panel: quantitative analyses on mRNA of ERα, Bmi1 and p16^INK4a^. All data were obtained from three independent experiments and are shown by bars as means ± SD (^**,## or △△^P < 0.01, ^***^P < 0.001 when ERα, Bmi1 and p16^INK4a^ were compared with the NS group, respectively).

Taking another approach, three pairs of siRNAs against different sequences of ERα were synthesized and transiently transfected into MCF-7 cells, and after 72 h the effect of ERα silencing was confirmed by western blot. The level of ERα protein was markedly reduced by siRNA3 (Figure 5A). ERα depleted MCF-7 cells showed a decrease in expression of Bmi1, but expression of p16^INK4a^ increased as compared to the controls (NS group) (Figure 5B).

In summary, these results implied that ERα may specifically stimulate the functional expression of Bmi1.

### ERα up-regulated Bmi1 expression at the transcription level

As a classic steroid hormonal receptor, ERα generally regulates its target genes at the transcriptional level. The sequences of the *Bmi1* promoter were therefore retrieved and bio-informatically analyzed. The *Bmi1* promoter contains a series of GC-rich sequences close to its transcription start site, and several putative transcription factor elements including AP-1 (activator protein-1) and Sp-1 (specificity protein-1) in addition to one confirmed E-box (enhancer-box) [13,27,28], in which two putative half estrogen responsive elements (ERE) were found to overlap with the AP-1 and Sp-1 elements (Figure 1A). Various regions which encompassed the *Bmi1* up-stream sequences according to the database sequences were amplified and a series of luciferase reporters were generated, including pGL2-1200, pGL2-900, pGL2-460, pGL2-240 and pGL2-152 (Figure 1B).

With a dual reporter system, MCF-7 and MDA-MB-231 cells were transiently transfected with pGL2-1200, pGL2-900, pGL2-460, pGL2-240 or pGL2-152 together with a pRL-SV-Renilla luciferase reference vector. As expected, MCF-7 and MDA-MB-231 cells showed significantly different reporter activities (Figure 1C). With treatment of E2 (10^−8^ M), the reporter activity of the *Bmi1* promoter constructs was slightly increased in MCF-7 but not in MDA-MB-231 cells (Figure 1C). However, upon co-transfection of the luciferase reporters with pcDNA3.1-ERα into MCF-7 cells, there was an overall increase in transcription activity of the *Bmi1* promoter (Figure 1D). In order to observe the specificity of the effect of ERα, the *Bmi1* promoter reporters were transfected into ERα-restored MDA-MB-231 cells (231/ERα), and showed increased transcription activity as compared to empty vector-transfected MDA-MB-231 cells (231/vec) (Figure 1E). These results proved that ERα could activate the transcription activity of the *Bmi1* core promoter.

**Figure 6:**
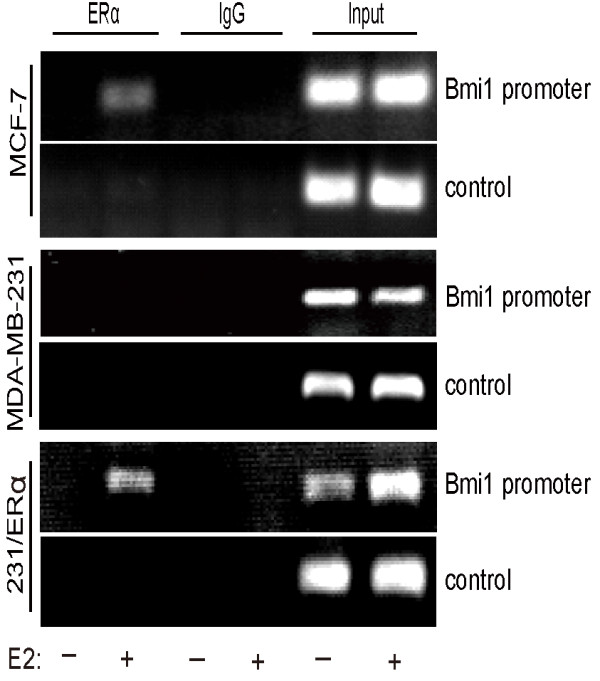
**Interaction of ERα and Bmi1 promoter.** MCF-7, MDA-MB-231 and 231/ERα treated or untreated with 10^−8^ M E2 for 12 h were used for Chromatin immunoprecipitation (ChIP) analysis of the human Bmi1 promoter as described in Methods. ERα: anti-ERα antibody; IgG: rabbit IgG. Bmi1 promoter: region (−327 bp to −172 bp), and control: region (−2647 bp to −2523 bp). PCR products were resolved on a 2% agarose gel.

We further tested for ERα binding on the *Bmi1* promoter in MCF-7, MDA-MB-231 and 231/ERα cell lines with ChIP. Following treatment of the cells with 10^−8^ M E2, DNA immunoprecipitated with anti-ERα antibody was amplified using *Bmi1* promoter primers to evaluate the interaction of ERα with the *Bmi1* promoter at −327 ~ −172 bp (Figure 6). Results confirmed that ERα can interact with the up-stream element of the *Bmi1* promoter.

### ERα-coupled Bmi1 regulatory pathway in breast cancer

To evaluate the functional role of the ERα-coupled Bmi1 regulatory pathway in breast cancer, the expression of p16^INK4a^ or cyclin D1 which are target genes of Bmi1 and ERα [29] respectively, was measured and their correlation with other indices of breast cancer was analyzed.

#### Down-regulation of ERα and Bmi1 correlated with aberrant expression of p16^INK4a^

In normal tissues adjacent to breast cancer, p16^INK4a^ was expressed only in nuclei of occasional cells (Figure 2), while p16^INK4a^ showed aberrant staining of tumor cells in 29.35% (27/92) of breast cancers, and this positive staining was generally present in both the nuclei and cytoplasm (Figure 2). Even in some cases, staining was present mainly in the cytoplasm with decreased or absent nuclear staining. Aberrant staining for p16^INK4a^ was found in 71.88% (23/32) of ERα negative cases out of a total of 92 cases of invasive carcinoma, compared to ERα positive tumors (6.67%, 4/60). Similarly, p16^INK4a^ was frequently expressed in progesterone receptor (PR) negative tumors (66.67%, 22/33) and was positive in only a small number of cases in the PR positive group (8.47%, 5/59). p16^INK4a^ expression showed a strong inverse correlation with ERα and PR expression status (*P <* 0.0001, *P <* 0.0001) (Table 1), indicating that aberrant expression of p16^INK4a^ is associated with loss of hormone receptors. Similarly, Bmi1 was positive in most of the p16^INK4a^ negative group (98.46%, 64/65), while Bmi1 negativity was found frequently with aberrant staining of p16^INK4a^ (66.76%, 18/27). There was a significant negative correlation of Bmi1 with p16^INK4a^ (*P <* 0.0001) (Table 1), demonstrating aberrant expression of p16^INK4a^ is associated with reduced Bmi1 expression.

To gain further insight into the pathologic implications of loss of ERα-coupled Bmi1 inducing abnormal p16^INK4a^ expression, the relationships between aberrant p16^INK4a^ expression and other factors in breast cancer such as HER2 and Ki-67 were analyzed. p16^INK4a^ expression was found in a majority of triple-negative breast carcinomas (TNBC) (85.71%, 12/14) and HER2-enriched carcinomas (68.75%, 11/16), whereas it was less frequent in Luminal-B type tumors (15.38%, 4/26) and was not found in Luminal-A tumor (0.00%, 0/28) or in Luminal-HER2-Positive tumors (0.00%, 0/8). p16^INK4a^ positivity in triple negative breast cancer and HER2-enriched subtypes showed a marked statistical difference from tumors in the other three groups (*P <* 0.0001) (Table 2). This specific distribution of aberrant p16^INK4a^ expression over the various molecular subtypes pointed strongly to a relationship with hormone receptor status.

The Ki-67 index is a chief factor for distinguishing the Luminal-A and Luminal-B subtypes, so the relationship of p16^INK4a^ and Ki-67 expression was analyzed. For this purpose, cases were classified into four Ki-67 expression index groups which included 0-13%, 14%-29%, 30%-49% and 50-100%. The positivity rates of p16^INK4a^ in these four groups were 13.16% (5/38), 6.25% (1/16), 38.89% (7/18) and 70.00% (14/20), respectively. This result demonstrated strong correlation of aberrant expression of p16^INK4a^ with the Ki-67 index (*P <* 0.0001) (Table 1).

#### Expressions of ERα and Bmi1 correlated with activated cyclinD1

Since cyclin D1 is a target of ERα [30], its expression was analyzed with respect to either ERα or Bmi1. Cyclin D1 was also positive more frequently in the ERα positive group (90.00%, 54/60) (Figure 2), as compared to the ERα negative group (50.00%, 16/32). Similarly, positive cyclin D1 was also found in most cases positive for Bmi1 (86.30%, 63/73), while only a few cases of cyclin D1 were found in the Bmi1 negative group (36.84%, 7/19). These results demonstrated a positive correlation between ERα, or Bmi1 and cyclin D1 (*P <* 0.0001, *P <* 0.0001). In ad–dition, results showed that cyclin D1 positive cases predominated in the p16^INK4a^-negative group (84.62%, 55/65), as compared to 55.56% (15/27) of cases which were p16^INK4a^-positive, showing an inverse correlation with aberrant expression of p16^INK4a^ (*P =* 0.003) (Table 3).

**Table 3:** **Correlation of Cyclin D1 or pRB with ERα, Bmi1 and p16**^**INK4a **^**expression in breast cancer**

	**Cyclin D1**		***P *** **value**	**pRB**		***P *** **value**
	**–**	**+**		**–**	**+**	
**ERα**			**<0.0001**			0.465
–	16	16		20	12	
+	6	54		42	18	
**Bmi1**			**<0.0001**			0.659
–	12	7		12	7	
+	10	63		50	23	
**p16**^**INK4a**^			**0.003**			0.284
–	10	55		46	19	
+	12	15		16	11	
**CyclinD1**			**--**			0.257
–	–	–		17	5	
+	–	–		45	25	

Bold face representing significant data (P<0.05).

#### The aberrant expression of p16^INK4a^ or activated cyclin D1 did not correlate with pRB status

Phosphorylated RB (pRB), which is the inactivated form of the RB protein was analyzed, and there were no obvious differences in positive p16^INK4a^ staining in pRB negative versus pRB positive tumors (16/62, 25.81%; 11/30, 36.67%) (*P=* 0.284) (Table 3), indicating that p16^INK4a^ expression has no correlation with pRB expression in this system (Figure 2).

Although the positivity rate for pRB in cyclin D1 positive cases (35.71%, 25/70) was a little higher than that in cyclin D1 negative tumors (22.73%, 5/22), the difference between pRB expression and the cyclin D1 status was not statistically different (*P =* 0.257) (Table 3). In addition, pRB showed similar rates of positivity in both ERα-positive (30.00%, 18/60) and ERα-negative cases (37.50%, 12/32) (*P =* 0.465).

These data showed that neither aberrant p16^INK4a^ expression nor activated cyclin D1 correlated with pRB in these breast cancers.

Taken together, these findings show the ERα-coupled Bmi1 regulation pathway plays an important role in regulation of the genes and biological behavior of breast cancer. The expression of ERα usually increases both levels of Bmi1 and cyclin D1, while loss of ERα-coupled Bmi1 activity may result in aberrant p16^INK4a^ expression and is also generally consistent with a more aggressive breast cancer phenotype.

## Discussion

The role of Bmi1 and the related functional network that serves in regulation of normal cells and cancer cells have been studied extensively in recent years. However, regulation of its expression, and especially the mechanism of its up-regulation in cancers, has rarely been explored [14,15]. To date, only E2F1 and MYCN have been shown to be direct activators of *Bmi1* transcription in some kinds of cancers [31,32], and data regarding *Bmi1* in tumor biology are far from complete. The over-expression of Bmi1 and its 11-gene signature has been defined in breast carcinoma [18]. At the same time, investigation has shown that Bmi1 expression is positively correlated with ERα status in breast cancer [33-35]. However, the direct interaction between these molecules has not been evaluated.

ERα is extremely important in tumorigenesis in female sex organs, and its signaling pathway has thus been extensively investigated [2,4]. As a classic nuclear receptor, it translocates into the nucleus upon binding of estrogen to dimerized ERα, and brings about activation of transcription of target genes via interaction with either ERE (estrogen responsive element) or other factors such as steroid receptor coactivator-1 (SRC-1), amplified in breast 1 (AIB1, also known as NCOA3) and E1A binding protein as well as p300/CREB binding protein (p300/CBP) [6,36,37]. The *Bmi1* promoter is a classic house-keeping gene as it possesses such features as a non-TATA box, and it has GC-rich sequences, putative AP-1 and Sp-1 elements, and a functional E-box, which has been identified to interact with several transcription factors in regulation of cell proliferation, stress and senescence [13,27,28]. Our experiments demonstrated that ERα can activate the transcription of *Bmi1* through directly interacting with its promoter. However, it is noteworthy that the magnitude of E2-inducing *Bmi1* promoter activity appears to be much lower than that of E2-inducing Bmi1 protein levels in MCF7 cells. It is possible the Bmi1 promoter was weak in transcriptional activity in this study, as only low reporter activity was measured under basal conditions (Figure 1C). On the other hand, our study also revealed that over-expression of ERα markely stimulates transcription activity of the *Bmi1* promoter as compared to the addition of E2 (Figure 1D, C). This may imply that the level of endogenous ERα more effectively affects the *Bmi1* promoter than the concentration of cellular estrogen, which is consistent with the correlation found in the ERα status and the expression of Bmi1 in breast cancer. Although only two putative ERE elements were found to be embedded in the E-box and AP-1 consensus, it seemed that the upstream GC-rich sequences might be involved, since luciferase reporters spanning the −425 region were responsive to transfection of ERα. Such results are similar to those which have been found in many ERα response genes, in which the GC-box, AP-1, −2 or Sp-1 are involved in ERα stimulation [36,37]. Despite the fact our investigation revealed that Bmi1 expression is determined by ERα status in breast cancer, it must be noted that nearly half of the cases with loss of ERα still expressed Bmi1 or cyclin D1 which is another ERα target gene [29,38]. This suggested the possibility that Bmi1 or cyclin D1 is induced mainly by ERα. However if there is no expression of ERα, Bmi1 may be induced by other factors such as E2F1 and MYC [31,32], both of which are usually expressed in cancers.

p16^INK4a^-cyclinD1/RB consists of machinery for regulation of cell cycle progression. Several observations have suggested that inactivation of RB in the genome or in the phenotype can lead to abnormal expression of p16^INK4a^ or cyclin D1, and promote cell cycle G1-S transition, resulting in sensitivity to loss of inhibition by ERα in cancer cells [39,40]. Therefore, the disruption of p16^INK4a^-cyclinD1/RB is believed to be a mechanism of resistance to endocrine therapy. However, our investigation showed that the expression of p16^INK4a^ and cyclin D1 is largely dependent on the activity of the ERα-coupled Bmi1 regulatory machinery, but not on RB status. In fact, although a preliminary report implied that p16^INK4a^/RB is abnormally expressed in breast cancer [33], recent studies demonstrated that over-expression of p16^INK4a^ is indicative of a more undifferentiated malignant phenotype in mammary carcinoma, such as the basal-like phenotype, in which ERα is generally negative [34].

p16^INK4a^ is a well known tumor suppressor and loss of its activity has been found widely in many kinds of human cancers [41]. However, its aberrant expression, in which p16^INK4a^ not only over-expresses but also changes its subcellular distribution from nuclear to cytoplasmic, has also been found in some types of cancer, especially in precancerous cervical lesions and cancers [42]. In fact the aberrant expression of p16^INK4a^ has become a pathologic indicator of high tumor grade in cervical precancerous lesions [43]. As p16^INK4a^ is a cyclin-dependent kinase inhibitor which is closely related to RB status, the aberrant expression of p16^INK4a^ is usually considered to be the result of compensation for RB inactivation, which occurs frequently in HPV-related cervical carcinogenesis [41,42]. Nevertheless, in this study we found a positive relationship between p16^INK4a^ and Bmi1 instead of RB. In fact, p16^INK4a^ has long been understood to be a target of Bmi1, and a recent study has shown multiple Bmi1-interactive elements in the upstream of *p16*^*INK4a*^[44]. These results indicate that in contrast to cervical neoplasia, the disruption of the ERα-Bmi1 pathway is most likely a primary cause of aberrant p16^INK4a^ expression in breast cancer. In addition, aberrant expression of p16^INK4a^ may be a marker for ERα negative breast cancers such as the TNBC and HER2-enriched subtypes, and may also be useful in distinguishing HER2-enriched from Luminal-B-HER2 positive breast tumors.

This study also demonstrates a positive relationship between ERα-Bmi1 and cyclin D1 in ERα-positive breast cancer. However, direct interaction exists only between cyclin D1 and ERα rather than Bmi1, and the expression of cyclin D1 in breast cancer probably reflects the regulatory effect of ERα. Therefore, the distribution of cyclin D1 in the various tumor subtypes was consistently found to be correlated with ERα status.

However, considering the heterogeneity of breast cancer, the clinical cohort in this described research is relatively modest. Therefore, further investigation is necessary.

Nevertheless, more attention should be paid to the discovery that ERα regulated expression of Bmi1, which could be a challenge to the general opinion of Bmi1 for its crucial role in regulating self-renewal of stem cells or cancer stem cells [13-15]. However, in a comprehensive analysis of Bmi1, Pietersen et al. also reveals a more intrinsic role of Bmi1 in development and homeostasis of mammary glands [45]. They demonstrate that Bmi1 expresses especially highly in luminal cells, which usually express hormone receptors. And knock-out of Bmi1 affects differentiation and proliferation of mammary stem cells but not reduces their number. In addition, loss of Bmi1 can induce premature lobuloalveolar differentiation, indicating that Bmi1 also affects more committed cells of mammary gland [45]. Combining the data with our results, we believe that there should be a close functional relationship between ERα and Bmi1, which also be crucial in regulation of breast cancers, especially in luminal-type carcinomas. Therefore, the exact role of ERα coupled Bmi1 pathway in breast cancer need to be further explored.

## Conclusions

This investigation revealed a regulatory relation between Bmi1 and ERα, and demonstrated an ERα-coupled Bmi1 signaling pathway in breast cancer. These results further reinforced the critical nature of the role of ERα in the development and treatment of breast cancer, and successful identification and description of ERα associated molecular events will provide a greater foundation for prevention and therapy of breast cancer.

## Abbreviations

ERα: Estrogen receptor α; RB: Retinoblastoma protein; CIN: Cervical intraepithelial neoplasia; HPV: Human papilloma virus; PcG: Polycomb group; PRC1: Polycomb repressor complex 1; IHC: Immunohistochemistry; WB: Western blot; E2: 17β-estradiol; OHT: Hydroxytamoxifen; SiRNAs: Small interfering RNAs; ChIP: Chromatin immunoprecipitation; PR: Progesterone receptor; TNBC: Triple negative breast cancer; ERE: Estrogen responsive elements.

## Competing interests

The authors declare that they have no competing interests.

## Authors’ contributions

BZ, HW and HL conceived and designed the experiments. HW, BZ and HL performed experiments and drafted the manuscript. HW, BZ, XL, JZ, HZ, JM, LH, WH and MAM interpretated and analyzed the data. XL, YZ, HZ and SZ contributed reagents/materials/analysis tools. All authors have read and approved the final manuscript.

## Pre-publication history

The pre-publication history for this paper can be accessed here:

http://www.biomedcentral.com/1471-2407/14/122/prepub

## Supplementary Material

Additional file 1: Table S1 Clinical information of patients. **Table S2.** Primary antibodies used in this study. Click here for file
