# Supplementary material for: Estrogen receptor α-coupled Bmi1 regulation pathway in breast cancer and its clinical implications
Source: BMC Cancer. 2014 Feb 24;14:122. doi: 10.1186/1471-2407-14-122 (PMC3939403; doi:10.1186/1471-2407-14-122)
Supplement: Additional file 1: Table S1 — Clinical information of patients. Table S2. Primary antibodies used in this study. [file 1471-2407-14-122-S1.doc]

**Additional file 1**

**Table 1 Clinical information of patients**

|  |  | n | % |
| --- | --- | --- | --- |
| Tumor | T1 | 60 | 65.22 |
|  | T2 | 30 | 32.61 |
|  | T3 | 2 | 2.17 |
|  | T4 | 0 | 0 |
| Nodus | － | 60 | 65.22 |
|  | ＋ | 32 | 34.78 |
| Histological typing | Invasive ductal carcinoma | 92 | 100.00 |
| Grade | I | 23 | 25.00 |
|  | II | 35 | 38.04 |
|  | III | 34 | 36.96 |
| Menopausal state | Pre-menopause | 50 | 54.35 |
|  | Post-menopause | 42 | 45.65 |

**Table 2 Primary antibodies used in this study**

| Antibodies | Host | Clone | Dilution | Source |
| --- | --- | --- | --- | --- |
| ERα | Mouse, monoclonal | 1D5 | Prediluted (IHC) | Dako |
|  | Rabbit, monoclonal | EP1 | 1:300 (WB) | Epitomics |
| PR | Mouse, monoclonal | PgR 636 | 1:100 (IHC) | Dako |
| HER2 | Rabbit, monoclona**l** | 4B5 | Prediluted | Roche |
| Ki-67 | Mouse, monoclonal | MIB-1 | 1:100 (IHC) | Dako |
| Cyclin D1 | Rabbit, monoclonal | SP4 | Prediluted | Epitomics |
| pRB (pT356) | Rabbit, monoclonal | EPR2153AY | 1:50 (IHC) | Epitomics |
| Bmi1 | Mouse, monoclonal | 1.T.21 | 1:150 (IHC) | Abcam |
|  |  |  | 1:1000 (WB) |  |
| p16^INK4a^ | Mouse, monoclonal | G175-405 | 1:50 (IHC) | Dako |
|  |  |  | 1:300 (WB) |  |
| β-actin | Mouse, monoclonal | 6G3 | 1:5000 (WB) | Roche |
